# Bilateral thoracotomy for horseshoe lung complicated with severe aspergilloma: a case report

**DOI:** 10.1186/s40792-024-01853-6

**Published:** 2024-04-08

**Authors:** Chihiro Kagohashi, Yasuyuki Kurihara, Mariko Hanafusa, Kenichi Okubo

**Affiliations:** 1 Department of Thoracic Surgery, Japanese Red Cross Musashino Hospital, 1-26-1, Kyonan-Cho, Musashino-Shi, Tokyo, 180-8610 Japan; 2 Department of Thoracic Surgery, Tokyo Medical and Dental University, 1-5-45, Yushima, Bunkyo-Ku, Tokyo, 113-8519 Japan

**Keywords:** Horseshoe lung, Aspergilloma, Bilateral thoracotomy

## Abstract

**Background:**

Horseshoe lung is a rare congenital malformation in which the lung protrudes from the mediastinum to the other side. Owing to the high frequency of other fatal cardiovascular complications, it is often diagnosed in childhood and rarely unnoted until adulthood. We report a case of horseshoe lung in an older patient who underwent thoracotomy.

**Case presentation:**

The patient was a 69-year-old man with chronic obstructive pulmonary disease (COPD) and a history of heavy smoking. The patient was admitted to the hospital because of acute exacerbation of COPD. Computed tomography revealed horseshoe lung and pulmonary sequestration with pneumonia. This was the first time that he was diagnosed with horseshoe lung; however, he had been treated for pneumonia multiple times before. Surgery for the horseshoe lung was recommended; however, the patient declined it because his symptoms of acute COPD exacerbation were relieved by medication. *Aspergillus* infection of the horseshoe lung led to frequent bloody sputum, and the patient’s respiratory condition gradually worsened. Two years after the initial diagnosis, the patient decided to undergo the surgery. Surgery was performed in the order of left and right thoracotomies, with posterolateral thoracotomies performed bilaterally. Surgery was difficult because of strong adhesions around the inflamed lung; however, the lung was removed in one lump. The patient was extubated on postoperative day (POD) 1, and rehabilitation was initiated. His high sputum volume caused postoperative pneumonia, and the patient was again placed on a ventilator on POD 9. He underwent open-window surgery for concomitant pyothorax. The patient was weaned off the ventilator when the inflammation improved and was discharged on POD 133. The patient lived at home, developed severe pneumonia 4 months later, and died of respiratory failure.

**Conclusion:**

Pulmonary sequestration and horseshoe lungs are congenital malformations that require surgery. The selection of the optimal time for surgery is important.

## Background

Horseshoe lung is a rare congenital malformation in which a band of pulmonary parenchyma is formed, extending between the right and left lungs. Horseshoe lung causes other malformations, such as pulmonary sequestration [1, 2]. It is usually diagnosed in childhood because of severe respiratory and cardiovascular complications; however, there are few reports in which the disease was not diagnosed until adulthood. We report a case of horseshoe lung with pulmonary sequestration discovered only in old age and subsequent surgical treatment with bilateral thoracotomy.

## Case presentation

**Fig. 1:**
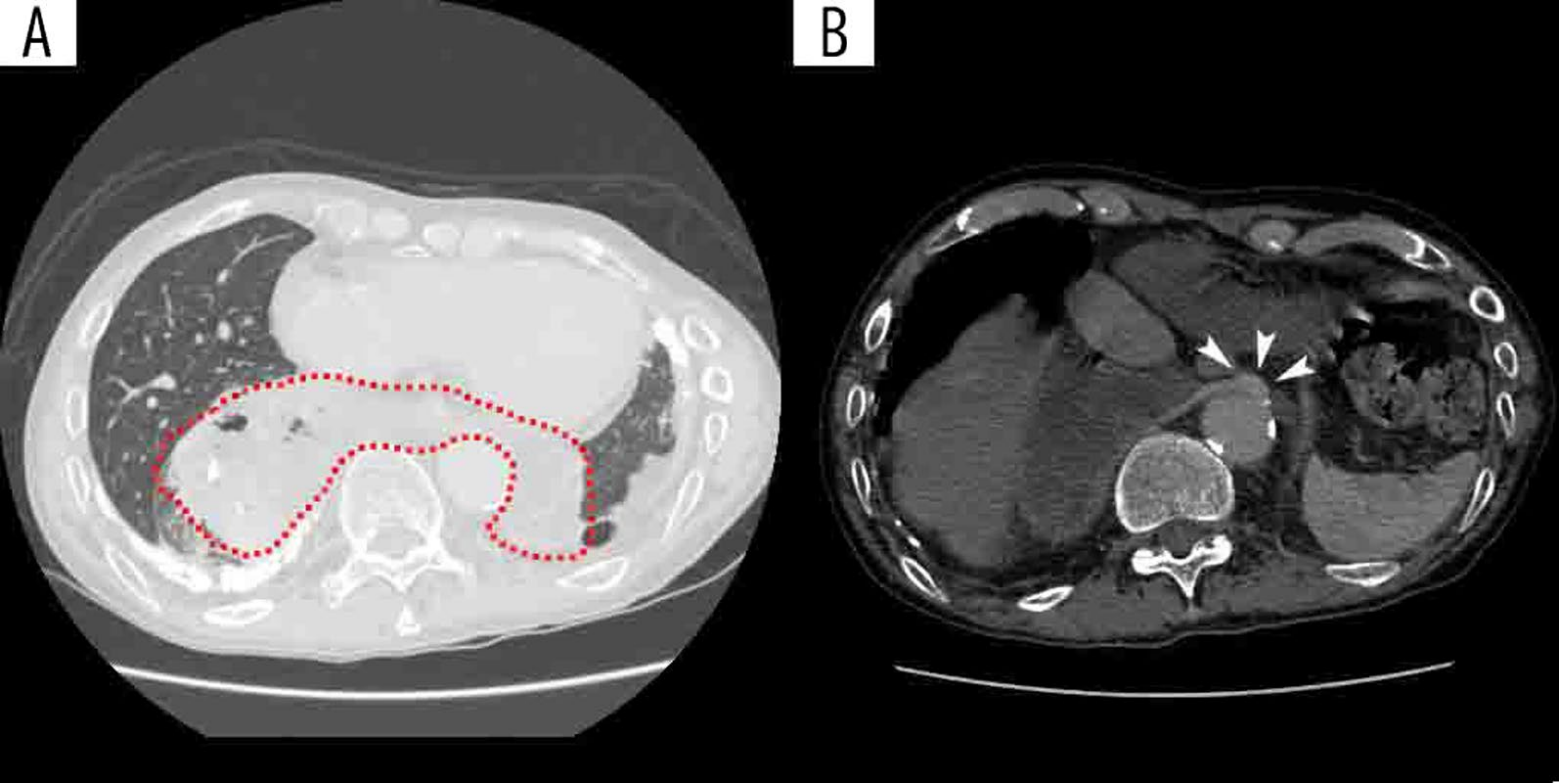
**A** Computed tomography showing the protruding lung from the right to the left side in front of the descending aorta and behind the esophagus (dotted line). **B** An abnormal blood vessel from the descending aorta to the lung is confirmed (arrow)

**Fig. 2:**
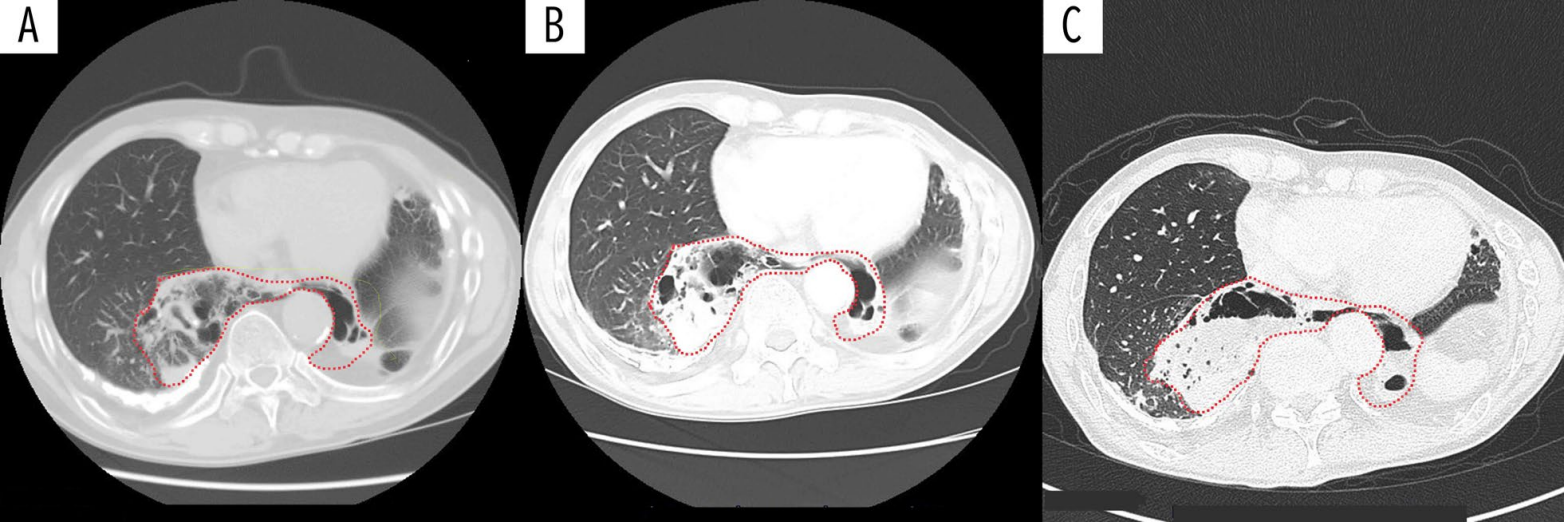
Computed tomography at 5 years (**A**), 3 years (**B**), and 1 year (**C**) before surgery. The horseshoe lung (dotted line) became rough because of repeated infections

A 69-year-old man was referred to our hospital with acute exacerbation of chronic obstructive pulmonary disease (COPD). Computed tomography revealed pneumonia in the right lower lobe and extralobular sequestration of the left lower lobe. His right lower lobe had a normally bifurcated bronchus and pulmonary arteriovenous system, and part of the lung parenchyma protruded beyond the mediastinum into the left thoracic cavity, in front of the vertebral body (Fig. 1). Abnormal vessels from the descending aorta flowed into the protruding lung. The protruding lung was distinguished from the normal left lower lobe by the pleura and was, therefore, in a state of left lower pulmonary sequestration. The patient was diagnosed with a congenital malformation of the horseshoe lung with pulmonary extralobular sequestration. Although the patient had been treated for pneumonia multiple times before, this was the first time a pulmonary abnormality was noted (Fig. 2). After the treatment was completed, we suggested surgery to remove the malformed lung. However, the patient declined because his symptoms were alleviated with medication. Over the next 2 years, the horseshoe lung repeatedly developed pneumonia. One year later, he was hospitalized for pneumonia, at which time *Aspergillus* was detected in his sputum for the first time. We determined that he had complicated chronic pulmonary aspergillosis. From this point on, he started taking itraconazole. He then experienced hemoptysis and underwent bronchial artery embolization twice; however, the hemoptysis was not completely cured. After repeated episodes of pneumonia and hemoptysis, his medicine was switched from itraconazole to voriconazole 3 months before the surgery. His pulmonary function gradually deteriorated owing to lung destruction. Home oxygen therapy was initiated 1 year later. Finally, at the age of 71 years, the patient requested surgery after a discussion with medical personnel and was referred to our department.

At that time, the patient experienced frequent phlegmatic cough, with hemoptysis. He was receiving home oxygen therapy and required 2 L of oxygen at rest and 3 L during exertion. The vital capacity (1.27 L) and forced expiratory volume in 1 s (0.80 L) were measured, both of which were worse than those measured 2 years before. The surgical indication was discussed at a multi-professional conference, and we concluded that the removal of the swollen lung could alleviate the symptoms and improve the respiratory condition; thus, we decided to perform the surgery. One week before the surgery, the patient was hospitalized and underwent respiratory rehabilitation. Blood tests just prior to surgery showed that his white blood cell count was 8500 and CRP, 0.34.

**Fig. 3:**
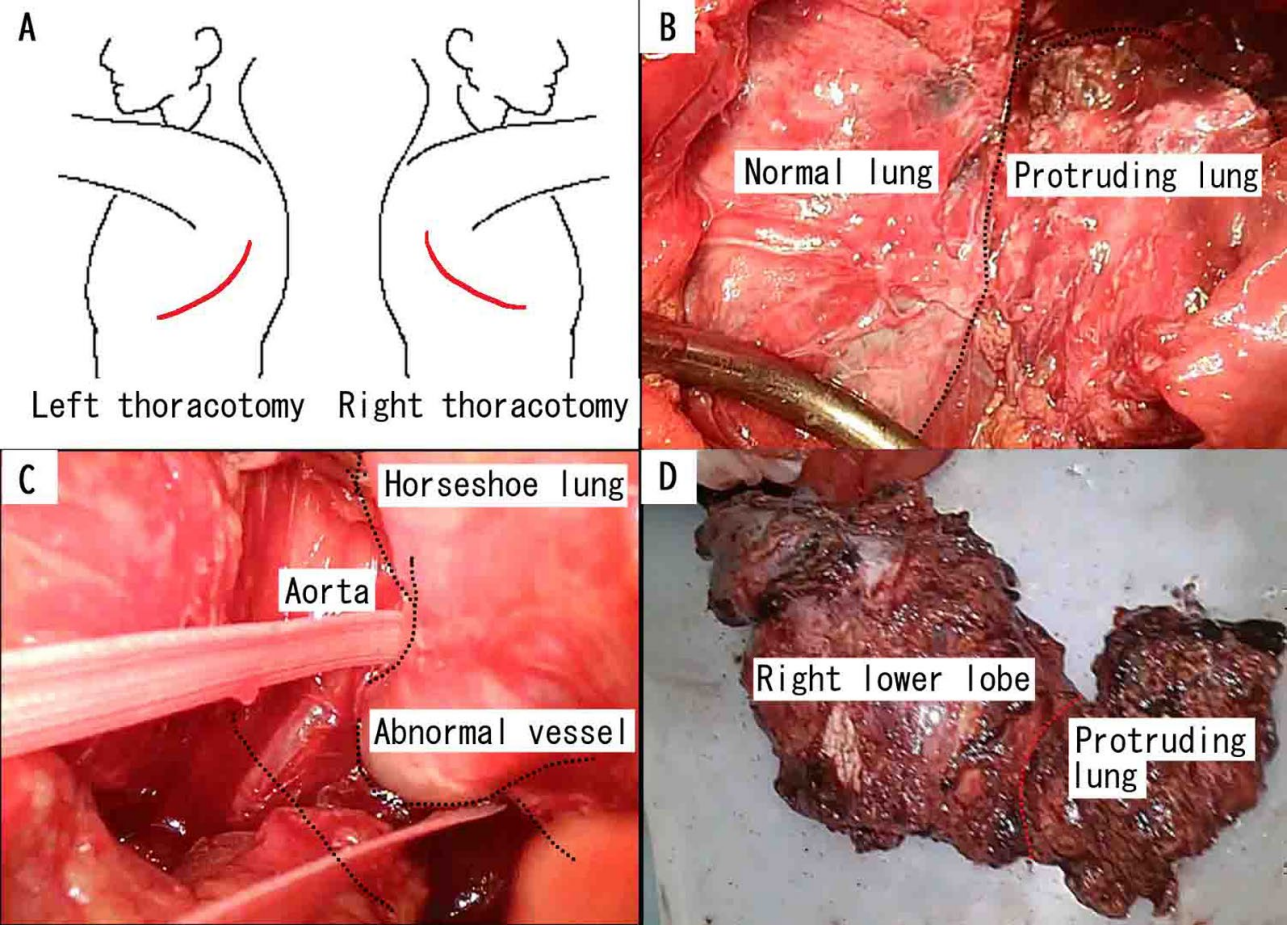
Surgical findings. **A** Skin incision lines. **B** From left thoracotomy, we can identify the normal and protruding lung. **C** Abnormal blood vessels during left thoracotomy. **D** Removed lung

Surgery was performed in the following order: left thoracotomy, followed by right thoracotomy, with posterolateral thoracotomy performed bilaterally (Fig. 3A). From the left thoracic cavity, we observed a normal left lower lobe and extralobular sequestration. There was dense and extensive adhesion; therefore, we carefully detached the extralobular sequestration from the chest wall, normal left lower lung, and diaphragm (Fig. 3B). The sequestration lung extended continuously to the right thoracic cavity between the vertebral body and esophagus. An abnormal blood vessel flowing into the sequestration from the descending aorta was identified from the left thoracic cavity and excised using automatic sutures (Fig. 3C, D). A right lower lobectomy was performed via a right thoracotomy. Although the degree of adhesion was even worse in the right thoracic cavity, we successfully removed the horseshoe lung without breaking the capsule. The operating time was 8 h and 39 min and blood loss was 1600 ml, and he received a blood transfusion (red cell concentrate, 4 units; fresh frozen plasma, 4 units).

The patient was extubated on postoperative day (POD) 1, and rehabilitation was initiated. However, he had acute exacerbation of COPD and required a ventilator on POD 9. *Aspergillus*, which caused empyema, was detected in the right pleural effusion. A right open-window thoracostomy was performed on POD 46. The patient’s respiratory condition improved thereafter, and he was weaned off the ventilator on POD 91. The patient was discharged after thoracostomy on POD 133. Antifungal medications were continued postoperatively.

At the time of discharge, he received home oxygen therapy (1 L at rest and 3 L during exertion). The patient’s oxygen requirements were almost the same as those before admission. The subjective symptoms of bloody sputum improved. He was still able to walk several hundred meters, but his activities of daily living were reduced compared with those before admission. He recovered at home but developed severe pneumonia 4 months later. The patient was hospitalized again and received antibiotics and antifungal drugs. However, despite extensive pneumonia treatment, he died of respiratory failure.

## Discussion

The horseshoe lung was first described by Spencer in 1962 [1, 2]. Similar conditions were reported by French anatomists in the nineteenth century [2]. To date, approximately 40 cases have been reported. Horseshoe lung is often diagnosed during childhood or at a young age. Prenatal diagnoses have also been reported [3]. Most patients require lung resection early in their lifetime because of other life-threatening cardiothoracic anomalies, such as scimitar syndrome (a subtype of partial anomalous pulmonary venous connection); lung hypoplasia; or bronchial, pleural, and diaphragm malformations [4, 5]. Pulmonary sequestration has also been reported. Although the surgical procedure may be complicated by the need for surgery for other malformations, we did not find any case reports of infection or adhesions being a problem because the surgeries were performed at a young age.

In this case, because we examined his chest images only from his advanced age, there was the possibility of acquired protrusion of the sequestrated lung into the left thoracic cavity with infection. However, we concluded that the lung protrusion was congenital due to his CT images were very similar to those of horseshoe lung cases in young patients. The protruding lung was large and also unlikely to be acquired protrusions thorough the mediastinal isthmus. The patient did not have life-threatening cardiovascular complications, and we believe that this is why his horseshoe lung with pulmonary sequestration was not observed until the age of 69 years. To our knowledge, this is the oldest adult patient diagnosed with horseshoe lung [6–9].

Later in life, the patient experienced repeated bouts of pneumonia, and his respiratory condition gradually worsened. His recurrent pneumonia was probably caused by a tendency to accumulate sputum in his horseshoe lung and the associated fractionated lung. However, he was only treated for pneumonia associated with COPD until his advanced age.

After his anatomical abnormalities were recognized, surgery was discussed. However, 2 years passed without surgery, partly because pneumonia treatment worked to some extent Eventually, the sputum was found to contain *Aspergillus*, and the patient’s respiratory condition worsened, with repeated bloody sputum. A decision was made to perform surgery under high-risk conditions. Excision of the lung with roughening and infection was difficult because of severe adhesions. Large bilateral open thoracotomy was needed. Although we successfully removed the lung in one lump without damaging the pleura, there was also a high risk of relapse of *Aspergillus* infection after surgery, which unfortunately became a reality. In children and young patients, the horseshoe lung can often be completely removed using one-sided thoracotomy. However, in our case, we had to perform bilateral thoracotomy with a large incision because of dense and extensive adhesions. Surgical invasion might have delayed postoperative recovery.

The patient also developed an infection after surgery, and an open-window thoracotomy was required. We believe that the patient’s immunity and respiratory condition had deteriorated because of his advanced age. Two years had passed since the malformation was noted until surgery. The respiratory function decline owing to *Aspergillus* infection during these 2 years may have caused postoperative respiratory complications, leading to death.

We believe that there was no way to fundamentally improve his condition other than by performing surgery, but it remains questionable whether the timing of surgery was correct. We wish that we had been able to diagnose him with imaging and recommend surgery before his horseshoe lung became aspergilloma. Therefore, it is important to determine the optimal timing of surgery.

## Conclusion

Pulmonary sequestration and horseshoe lungs are congenital malformations requiring surgery. The selection of the optimal time for surgery is important.

## Data Availability

All data analyzed during this study are included in the patient’s medical records in Japanese Red Cross Musashino Hospital.

## Abbreviations

COPD: Chronic obstructive pulmonary disease
POD: Postoperative day
